# Iron- and Neuromelanin-Weighted Neuroimaging to Study Mitochondrial Dysfunction in Patients with Parkinson’s Disease

**DOI:** 10.3390/ijms232213678

**Published:** 2022-11-08

**Authors:** Benjamin Matis Pizarro-Galleguillos, Liesa Kunert, Norbert Brüggemann, Jannik Prasuhn

**Affiliations:** 1 Facultad de Medicina, Universidad de Chile, Santiago 8380453, Chile; 2 Institute of Neurogenetics, University of Lübeck, 23588 Lübeck, Germany; 3 Department of Neurology, University Medical Center Schleswig-Holstein, Campus Lübeck, 23562 Lübeck, Germany; 4 Center for Brain, Behavior, and Metabolism, University of Lübeck, 23562 Lübeck, Germany

**Keywords:** Parkinson’s disease, mitochondria, iron, neuromelanin, neuroimaging, magnetic resonance imaging (MRI), magnetic resonance spectroscopy imaging (MRSI)

## Abstract

The underlying causes of Parkinson’s disease are complex, and besides recent advances in elucidating relevant disease mechanisms, no disease-modifying treatments are currently available. One proposed pathophysiological hallmark is mitochondrial dysfunction, and a plethora of evidence points toward the interconnected nature of mitochondria in neuronal homeostasis. This also extends to iron and neuromelanin metabolism, two biochemical processes highly relevant to individual disease manifestation and progression. Modern neuroimaging methods help to gain in vivo insights into these intertwined pathways and may pave the road to individualized medicine in this debilitating disorder. In this narrative review, we will highlight the biological rationale for studying these pathways, how distinct neuroimaging methods can be applied in patients, their respective limitations, and which challenges need to be overcome for successful implementation in clinical studies.

## 1. Introduction

Parkinson’s disease (PD) is the second most common neurodegenerative disorder affecting millions worldwide [1]. For still unknown reasons, PD is the fastest-growing neurodegenerative disorder providing a significant burden to aging societies [2]. Besides recent advances in elucidating the underlying disease mechanisms, disease-modifying therapies (DMTs) are currently not available, and the treatment of patients with PD (PwPD) mainly relies on symptomatic relief [1]. One major obstacle to the development of DMTs is the complex pathophysiology of the disorder, involving a tapestry of molecular events, finally leading to the degeneration of dopaminergic (DA) midbrain neurons and other neuronal populations [3]. Several hypotheses have been postulated in the past concerning the deposition and aggregation of alpha-Synuclein (aSyn), mitochondrial dysfunction, disturbances of DA metabolism, the involvement of metal ions, impaired autophagy/lysosomal pathways, and neuroinflammation, among other mechanisms [4]. These proposed mechanisms are highly interconnected and may only be present in the individual disease course at a particular time point. In addition, there is a complex interplay present between genetic and environmental factors in PD pathophysiology. The elucidation of monogenic causes of PD led to the discovery of lead mechanisms (e.g., aSyn deposition by the identification of disease-causing mutations in the *SNCA* gene) that have also fostered the elucidation of complex genetic causes explaining the missing heritability in some cases [3]. The idea of mitochondrial dysfunction in PD pathophysiology was originally derived from environmental studies, where potent inhibitors of complex I of the ETC have been identified as causing PD (i.e., MPTP). Interestingly, the later discovery of monogenic causes (*Parkin* and *PINK1*) has also stressed mitochondrial dysfunction as a key disease mechanism in PD [5]. Accordingly, PD may serve as a model disease for individualized pathway-based therapies. This molecular complexity is a significant challenge for conceptualizing clinical trials for evaluating future DMTs [3,5,6]. To date, long interventional periods to consider neuroprotective therapies are necessary to observe any significant effect. It would, therefore, be desirable to identify the most suitable study participants to test a candidate drug based on the predominant individual disease mechanism. However, distinct prerequisites, i.e., the concomitant development of pathophysiology-orientated biomarkers, are necessary to facilitate such individualized treatment decisions [7]. Neuroimaging methods provide a unique window to the human brain to gain pathophysiological insights. In particular, MRI-based methods to map iron deposition, neuromelanin (NM) levels, and surrogate markers of mitochondrial dysfunction (namely disturbances of oxidative phosphorylation, OXPHOS, and oxidative stress) have been on the rise and allow for deepened insights into the individual disease manifestation, predominant disease mechanisms, and disease progression [7,8,9]. However, methodological heterogeneity and the lack of multimodal studies hinder the translation into clinical practice.

### The Scope of This Review

With this review, we illustrate how iron- and NM-weighted neuroimaging have been used to date in PwPD, how methodological considerations can improve the clinical applicability, and how these neuroimaging modalities can be combined for the study of mitochondrial dysfunction in PwPD.

## 2. Main Body

### 2.1. Iron Dyshomeostasis as a Disease Mechanism in PD

Iron is a metal, and besides being one of the most common elements on our planet, it is also essential for almost all living organisms. In this case, it is necessary for several metabolic processes, including oxygen transportation and DNA synthesis [10,11]. Iron is the most abundant non-diamagnetic element in the human brain and is stored as *hemosiderin-6* or *ferritin* [12]. In the central nervous system (CNS), the role of iron in OXPHOS, myelin production, and neurotransmitter metabolism have manifold pathophysiological implications [13]. Iron can harm CNS tissues due to hydroxyl radical production, which causes the oxidation and modification of different biomolecules. A complex network of regulatory proteins and signaling pathways is needed to maintain iron homeostasis in the brain [14].

Interestingly, iron is not primarily enriched in the substantia nigra (SN) pars compacta (SNpc); its presence is more relevant to functions related to the SN pars reticulata and the pallidum. Additionally, it is present in the red nucleus, the striatum, the dentate nucleus, and the subthalamic region [15]. Even though the deposition of iron is not specific to neurons in general, the neurons of the dopaminergic system are particularly vulnerable due to their high energy demand and DA metabolism [16]. Accumulation of iron is not necessarily related to disease but is also observed in normal aging. Here, different areas, such as the globus pallidus, the SN, the dentate nucleus, and the motor cortex, are predominantly involved. However, a more strongly pronounced deposition has been postulated in neurodegenerative disorders [17]. Subsequently, iron dysregulation has been proven to be implicated in several neurodegenerative disorders, such as Huntington’s disease, Alzheimer’s disease, motor neuron disease, and frontotemporal dementia [13,14,18,19]. Iron overload has also been implicated in the development of PD [18]. The SN is one of the first areas to exhibit abnormal iron deposition, but the putamen has also been shown to be affected in post-mortem studies. These findings align with MRI-derived findings that have been reviewed previously by Wang et al. (2016) [18]. However, whether increased iron deposition is either a cause or merely an epiphenomenon of PD remains elusive.

Further insights and the integration of multi-methodological knowledge on distinct iron pathways in PD are needed to elucidate this question [15]. Despite these yet unanswered questions, iron-targeted candidate drugs have been evaluated in clinical trials [20,21]. However, these clinical trials would need reliable biomarkers of pathophysiological underpinnings, stratification, and progression biomarkers for precision medicine. The contradictory results may be partly due to patients’ heterogeneity [22,23,24,25,26,27,28,29,30]. Interestingly, iron concentrations in the SNpc have been shown to be lower before the start of dopaminergic medication, increase throughout the disease course, and plateau at late disease stages. This observation led to the conclusion that the role of nigral iron deposition as an independent etiological factor may be doubtful [31]. One of the strategies to address these difficulties is to use objective biomarkers for early diagnosis and progression. Iron-weighted neuroimaging seems to be one of the most promising techniques, as peripheral iron measures are not well-correlated with brain iron levels [32]. In other neurodegenerative disorders (i.e., neurodegeneration with brain iron accumulation, NBIA), iron-weighted neuroimaging has already been used as a target engagement strategy for deferiprone (an iron-chelating drug that can cross the blood–brain barrier) [21].

### 2.2. The Complex Role of NM in PD Pathophysiology

Melanin is a generic name for a ubiquitous and heterogeneous biological pigment present among several phylogenetic kingdoms [33,34,35]. It is described as a heterogeneous macromolecule derived from the oxidation and subsequent polymerization of intermediate phenols and quinones [36]. Melanin can be identified in bacteria, fungi, plants, and animals [36]. Several functions are known, including visual communication, photoprotection, and antioxidative properties, among others [37]. According to its widespread distribution, melanin is found throughout an animal’s body, including in the skin, eyes, hair, inner ear, and brain [38]. In mammals, two main types of melanin are described: the brownish-like color eumelanin and the reddish-yellow-like color pheomelanin [39]. Both melanin pigments arise from *tyrosinase*-mediated oxidation of the common precursor, *L-tyrosine* and *L-DOPA* [40].

Additionally, a third type of melanin, NM, is described. NM is a dark-colored, complex, and insoluble pigment in different central nervous system cells, especially SNpc and locus coeruleus (LC) [41,42]. NM-containing neurons have also been described in other brain areas, such as the premotor cortex, the putamen, the hypothalamus, the medulla oblongata, and the cerebellum [43].

NM is a complex macromolecule consisting of pheo- and eumelanin, but also covalently bound lipids and peptides [42]. However, the biosynthesis pathways differ between NM and peripheral melanin [44]. Although the peripheral melanin pigment is relatively well understood, the production of NM is still the subject of ongoing research. Melanin production is driven by a biosynthetic pathway in which *tyrosinase* is the rate-limiting enzyme, whereas NM seems to result from spontaneous non-enzymatic DA oxidation [45,46]. Furthermore, NM is present not only in humans but also in the brains of mice, rats, dogs, horses, and monkeys [47]. Despite the presence of NM in several mammals, high NM concentration is only seen in the CNS of humans, which is consistent with the fact that the brain pigmentation of the areas above is not macroscopically observable in other animals [48,49]. Furthermore, the next-highest NM content can be found in animals in closer phylogenetic relation to humans [48,49]. These findings led to theories interpreting PD as a direct consequence of human evolution, additionally stressed by the fact that PD only naturally occurs in humans [48].

Additional functions of NM have been implied: these extend to redox activities, free radical scavenging, binding of different biomolecules, and the chelation of iron [36]. Surprisingly, NM has not only been shown to yield neuroprotective properties but can also be interpreted as a *double-edged sword*, promoting neurodegeneration [42]. Studies have shown that NM is a protective factor against *DA-quinones* and *reactive oxygen species (ROS)*, which occur within the biosynthesis of catecholaminergic neurotransmitters [36,50,51]. The metal-binding property of NM also exerts a neuroprotective effect by opposing the stress caused by *tyrosine-hydroxylase*- or *mitochondrial-cytochrome*-mediated Fe^3+^ release, especially in the SNpc [36,52]. However, an important and well-known fact is that the amount of NM in the cytoplasm of catecholaminergic neurons increases with normal brain aging [53,54,55]. The former could give us clues about the role of NM in normal aging, age-related motor abnormalities and neuropsychiatric illnesses [56]. In PwPD, NM-containing neurons consistently show degeneration, which exceeds the degree expected from normal aging. These regions include the SNpc, LC, and the dorsal motor nucleus of the vagal nerve, which are associated with characteristic motor and non-motor symptoms and signs of PD [46,57,58]. Several histopathological studies of these regions have shown how neuronal cell loss correlated well with the estimated NM loss, especially in the SNpc [59,60,61]. On the other hand, DA neurons in the *ventral tegmental area*, which are likely not or much less prone to neurodegeneration in PD, do not contain a significant amount of NM, supporting the role of NM in the neurodegenerative process of catecholaminergic SNpc neurons [62]. Recent evidence from rodent models stresses the important role of NM in PD, where *tyrosinase* overexpression in the SN of rats induced the age-dependent production of human-like NM in nigral DA neurons. The subsequent accumulation of human-like NM above a specific threshold was associated with a subsequent PD-like phenotype, Lewy body-like formation, and nigrostriatal neurodegeneration in rats [63]. In contrast, researchers have proposed the *NM hypothesis of PD*, trying to explain the occurrence of neuronal death resulting solely from the NM decrease in the DA neurons of the SNpc [47]. In this sense, measuring NM reduction as a proxy of catecholaminergic neuronal cell loss and an independent pathophysiological factor can aid in the in-depth understanding of PD.

NM is an effective metal chelator, hence it is the main iron compound found in catecholaminergic neurons. The function of NM is to trap iron and protect neurons from oxidative stress. The balance between iron, DA metabolism, and NM is crucial for cell homeostasis and can be disrupted under certain conditions [42,55]. In addition, NM has also been shown to initiate proinflammatory microglia through a *caspase-8*-dependent mechanism [64]. To further elucidate the interconnected nature of iron, NM-dyshomeostasis, and DA metabolism, not only in vitro or post-mortem studies are available [65]. Alterations of NM and its role in PD pathophysiology can be assessed in vivo through NM-sensitive MRI (NM-MRI). NM-MRI is a non-invasive technique that can also be applied in a clinical setting [66]. The latter is significant, as new DMTs are currently under research [67]. NM-MRI, among other MRI-based neuroimaging techniques, is promising due to its noninvasive nature and repeatability.

### 2.3. Mitochondrial Dysfunction at the Intersection of Iron- and NM-Related Pathways

**Figure 1:**
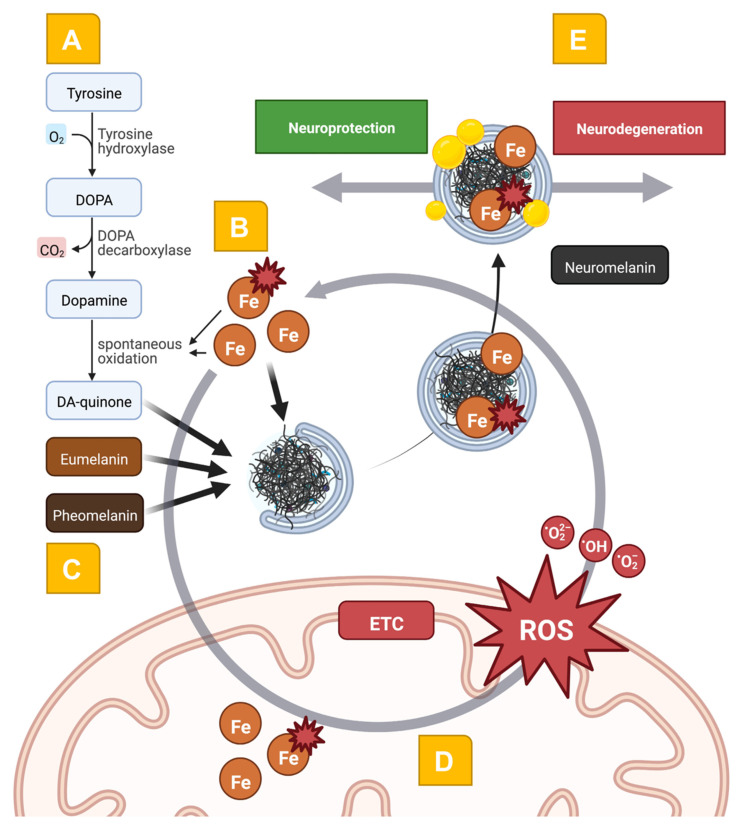
Schematic representation of the interwovenness of DA metabolism, iron, neuromelanin, and mitochondrial dyshomeostasis in the pathophysiology of PD. In **Panel** (**A**), the spontaneous oxidation of DA leads to the generation of the toxic DA-quinone. This process is further enhanced by the presence of ROS and a disbalance between ferrous and ferric iron (**Panel** (**B**)). The macromolecular organization of eumelanin and pheomelanin with DA-quinone leads to neuromelanin formation (**Panel** (**C**)). Here, macroautophagy-related mechanisms and the fusion with lysosomes and autophagic vacuoles form the complex macromolecule neuromelanin. Covalently bound lipids (depicted as yellow droplets) and peptides change the structure of neuromelanin over a lifetime, also in normal aging. In **Panel** (**D**), the exceeding import of iron and the improper assembly of iron-containing biomolecules (e.g., the ETC complexes) leads to bioenergetic disturbances and the generation of ROS. These mechanisms form a vicious cycle leading to neurodegeneration. Interestingly, the neuromelanin molecule has opposing roles in the pathophysiology of PD (**Panel** (**E**)): the complexation of damaging molecules serves as a neuroprotective mechanism via the reduction of oxidative stress. ETC: electron transport chain, Fe: iron, ROS: reactive oxygen species.

Mitochondrial dysfunction is a well-established concept in our current understanding of PD development [5]. However, *mitochondrial dysfunction* refers to many different pathophysiological aspects of mitochondrial homeostasis. These include the production of ATP via OXPHOS, coping with oxidative stress, metal ion and calcium homeostasis, the biosynthesis of macromolecules (e.g., electron transport chain, ETC, complexes), and apoptosis signaling, among others [5]. Interestingly, the underlying idea that mitochondria may play a prominent role in PD etiology was initially shown by environmental studies and later confirmed by the elucidation of monogenic PD forms caused by mutations in the *PINK1* and *Parkin* genes [68]. For a detailed review of several aspects of mitochondrial dyshomeostasis, we refer the interested reader to other articles covering this topic more in-depth [3,5,6]. Here, we will focus on the interconnectedness of mitochondrial dyshomeostasis with NM- and iron-related pathways (Figure 1). Mitochondria are responsible for regulating intracellular iron levels, their subcellular distribution, and complexation in their respective macromolecules [69].

The complexes of the electron transport chain (ETC) rely on iron-containing redox systems with iron–sulfur clusters and cytochromes with haem as prosthetic groups. The biosynthesis of haem groups and iron–sulfur clusters occurs partially in mitochondria. Altered iron import can lead to an iron overload into the mitochondria resulting in exceeding oxidative stress and impaired biomolecule assembly. These mechanisms lead to a self-sustaining loop of iron overload, excessive oxidative stress, and impaired mitochondrial bioenergetics [5]. Preclinical and human studies illustrated that mitochondrial dyshomeostasis leads to increased iron deposition in brain tissue [69,70].

Post-mortem studies proved that iron accumulation is predominantly present in the mitochondria. Some supporting evidence has been derived from studies in monkeys, where an increased iron deposition has been observed following *1-methyl-4-phenyl-1,2,3,6-tetrahydropyridine* (*MPTP)* treatment, an essential inhibitor of complex I in the ETC [71,72,73]. Different hypotheses have been stated further to explain the functional link between iron and mitochondrial dyshomeostasis; for example, one theory suggests that the inhibition of ETC complex I leads to an increase of oxidative stress, which substantially damages iron–sulfur clusters, their respective assembly into macromolecules, and enhances the excessive import of iron in the mitochondria [69]. Following this self-sustaining loop, the imbalance of Fe^2+^ and Fe^3+^ further increases oxidative stress. Decreased glutathione levels have illustrated another interconnectedness following iron deposition in the SNpc [74]. The abovementioned disease mechanisms can also be prompted towards the role of NM in PwPD: NM functions as a chelator by binding metal ions, especially the redox-active Fe^3+^ [41].

Nevertheless, a broader vision considers NM-related disease mechanisms as one factor contributing to the overall complex pathophysiology of PD among aSyn aggregation. This can be supported by the observation that aSyn also aggregates in NM-rich neurons, forming its precursors, pale bodies, near and in close physical association with NM [46,75]. Noteworthy, further research highlighted the potential role of iron as the pivotal molecule linking these two mechanisms [74]. Strikingly, NM-mediated iron release increases oxidative stress, leading to proteasomal and mitochondrial dysfunction [76,77]. Both disease mechanisms are connected to the DA metabolism and the further occurrence of oxidative stress [78]. Here, the formation of hydroxyl radicals by the Fenton reaction mediates the blockage of dopamine oxidation [79,80]. Interestingly, iron dyshomeostasis and neuroinflammation are directly linked. The increase of ROS via excessive iron overload in the mitochondria leads to the activation of proinflammatory signal cascades [81]. Blood–brain-barrier-crossing chelating drugs have been shown to rescue mitochondria from iron overload [20,82]. However, whether this approach may also restore mitochondrial homeostasis is currently unknown.

### 2.4. Pathophysiology-Orientated Neuroimaging

#### 2.4.1. Mapping Iron Dyshomeostasis

**Figure 2:**
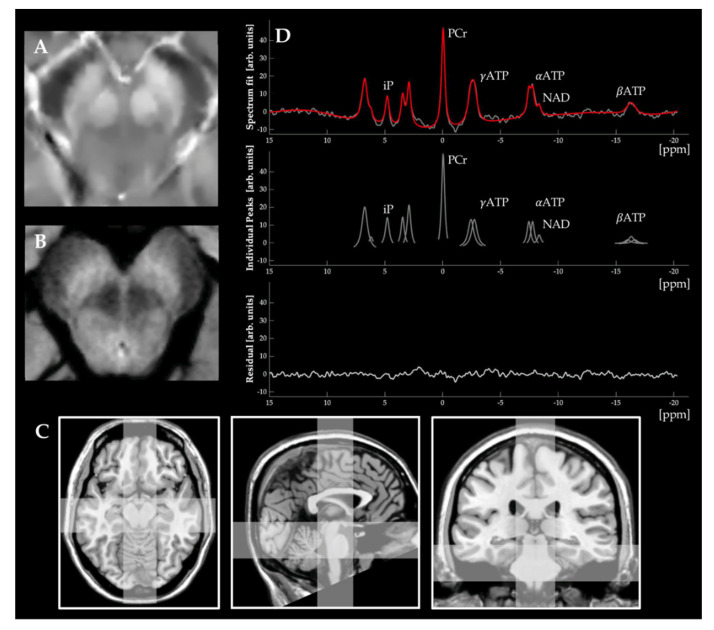
Examples of neuroimaging-based approaches to map iron-, NM-, and mitochondrial dysfunction. In **Panel** (**A**), we demonstrate an example of a QSM image of non-diseased midbrain (axial plane). In **Panel** (**B**), we demonstrate an example of an NM-weighted image of non-diseased midbrain (axial plane). Both images illustrate the spatial resolution and structural configuration of the SNpc, which can be achieved using clinical standard scanners (Siemens MAGNETOM Skyra, 3T, 64 channel head coil). In **Panel** (**C**), we exemplarily highlight the spatial resolution and voxel placement of ^31^P-MRSI measurements in the axial, sagittal, and coronal planes (from left to right). In **Panel** (**D**), the typical workflow of ^31^P-MRSI measurements can be seen. The acquired spectrum of a given voxel (upper panel, gray color) is interpreted by incorporating prior knowledge (spectrum fit, upper panel, red color). Here, the three different ATP peaks (αATP, βATP, and γATP) result from the three phosphate nuclei in an ATP molecule. Single peaks of known metabolites can be semi-quantified by calculating the area under the curve (middle panel). The residuals (lower panel) can be interpreted as a quality measure for the spectrum fit. ^31^P-MRSI: ^31^phosphorus magnetic resonance spectroscopy imaging. arb. units: arbitrary units. ATP: adenosine triphosphate. iP: inorganic phosphate. NAD: nicotinamide adenine dinucleotide. NM: neuromelanin. PCr: phosphocreatine. ppm: parts-per-million. QSM: quantitative susceptibility mapping.

Mapping iron deposition in PwPD is crucial for the early diagnosis and targeted future treatment [16]. Iron-weighted MRI is a promising tool to achieve this goal due to its noninvasive nature and availability and in vivo characterization of brain iron levels [83]. Advanced iron-sensitive imaging includes R2*/T2* relaxation imaging, susceptibility-weighted imaging (SWI), and quantitative susceptibility mapping (QSM, Figure 2, Panel A) [84]. The imaging contrast between different brain tissues depends mainly on the intrinsic relaxometry properties (T1 and T2 times) [85]. These can be assessed by the longitudinal and transverse relaxation times. T2* and its inverse value R2* (1/T2*) correspond to the transverse relaxation rate being an indirect measure of iron content without spin echo pulse-phase correction [86]. (Quantitative) Relaxometry is a technique that allows one to obtain these objective measures of tissue properties.

On the other hand, SWI and its quantitative counterpart QSM are techniques sensitive to magnetic inhomogeneity. This is possible due to subtle magnetic field shifts following variations of tissue magnetic susceptibility that alter the transverse relaxation times (T2) [87]. SWI/QSM can (semi-)quantify iron content, calcifications, and changes in venous oxygen saturation [88]. Therefore, in biological tissues, phase contrast is mainly given by iron (heme and non-heme iron), lipid, calcium, and myelin; however, in the deep gray matter, the phase contrast is primarily derived from iron [88]. In this way, several iron-sensitive MRI techniques have been developed, providing potential biomarkers for assessing iron deposition in PwPD and other neurodegenerative diseases. Table S1 gives an overview of current iron-weighted neuroimaging hardware setups, acquisition parameters, sequences, and study cohorts, including all high-quality studies that evaluated the diagnostic performance of iron-weighted neuroimaging in differentiating PwPD from HCs. The latter is necessary to further evaluate these acquisition schemes for studying mitochondrial dysfunction within the scope of iron-weighted neuroimaging.

#### 2.4.2. Iron-Weighted Neuroimaging in PwPD

One radiological sign has been defined: the ‘swallow tail sign’ represents the loss of signal intensity in T2-based sequences of nigrosome-1, one of the most affected regions of the SN, reflecting susceptibility artifacts of iron deposition [89,90]. However, the supposed diagnostic value is currently unclear [90,91,92,93].

In contrast to manually identifying radiological signs, (semi-)quantifying iron deposition in human brain tissue has become possible in vivo, providing more knowledge of its role in parkinsonian disorders [83]. The need for these techniques is underlined by the fact that peripheral iron levels and iron-regulatory proteins seem to not reflect the brain iron levels in PD, as was recently shown by a meta-analysis [94]. There is some evidence of MRI-derived iron measurements representing insights derived from post-mortem samples, which further stresses the biological meaning of these MRI modalities. Here, a meta-analysis suggested that R2* measures were higher in the SNpc and the red nucleus. These findings were also supported by SWI measures, which were significantly higher in caudate and putamen [18]. However, certain regions (e.g., the putamen) are more heterogeneous than the SNpc across different studies and modalities. Moreover, they did not match the pattern recognized in post-mortem samples. These differences may be, in part, consequences of other confounders, such as the presence of calcifications or microbleeds, image acquisition parameters, and post-processing, among others.

In a systematic review and meta-analysis on iron-weighted neuroimaging, the authors assessed the overall diagnostic performance of different iron-weighted neuroimaging modalities in differentiating PwPD from HCs [9]. The differentiation of PwPD and HCs can be considered a crucial prerequisite to defining methodological recommendations in the in vivo assessment of relevant disease mechanisms. Here, 22 articles, 1126 patients with PD, and 933 controls were included. Across the included studies, this technique achieved a pooled sensitivity of 92% (95% CI, 0.88–0.95) and a pooled specificity of 90% (95% CI, 0.81–0.95). These results expand and support the findings of another recent meta-analysis, which included ten studies that assessed the subjective visual analysis of iron-sensitive imaging; the group reported a pooled sensitivity of 98% and specificity of 95% [93]. Both studies included a variety of different iron-weighted neuroimaging methods and acquisition parameters. However, a third systematic review and meta-analysis also considered these systematic influences in their report [95]. Here, 46 studies fulfilled the inclusion criteria, considering 3155 PD patients and 1675 controls. The included meta-regression analysis illustrated that it seems reasonable to improve the reproducibility of iron-weighted neuroimaging by employing QSM at a static magnetic field strength of 3T, a voxel size of <0.6 mm^2^, a flip angle >15°, a slice thickness ≤1 mm, and a FOV specifically targeting the SNpc [9,95]. R2* and SWI seem less reliable for multi-center studies as there is high heterogeneity between studies. However, these results must be interpreted cautiously as the above-mentioned neuroimaging protocols are not completely standardized across different study sites [9]. Noteworthily, as a quantitative MRI method, QSM demonstrated a more robust and reproducible measure in studies that employed both R2* and QSM, mainly because there were no differences across different scanner manufacturers or hardware setups [95]. Thus, QSM is recommended by the authors for multicenter studies, which is supported by further studies summarized in another systematic review article [96]. Interestingly, phantom studies have illustrated that the differentiation of Fe^2+^ and Fe^3+^ can be achieved by relaxometry, which could provide unique insights into the disease mechanism of PwPD [97]. In conclusion, future studies have a raison d’être for combined iron-weighted neuroimaging modalities.

Iron-weighted neuroimaging has also been used in other studies to compare PwPD, their respective subtypes, and differential diagnoses [84]. In addition, there is some evidence that iron-weighted neuroimaging may differentiate patients with essential tremor (ET) from tremor-dominant PwPD [98]. The R2* values in the SNpc have been reported to be higher in PwPD with a gradual increase related to disease progression, suggesting a potential role in the longitudinal evaluation of PwPD [83,99,100]. These results are supported by several studies suggesting an early midbrain accumulation of iron only in the SNpc and the red nucleus across different imaging modalities [101,102,103,104,105]. Moreover, R2* values have also been correlated with distinct motor symptoms, e.g., with freezing of gait as the iron load increases [106]. Besides, brain iron deposition appears to be a surrogate marker of non-motor symptoms as well [107]. Iron-weighted neuroimaging identifies differences in iron deposition in patients with PD–dementia (PwPDD), showing higher magnetic susceptibility in PwPDD compared to PwPD in the left hippocampus and a negative correlation with cognitive function [108,109]. Results with similar biological meaning were reported, where a correlation of QSM-derived values with cognitive decline was observed [110].

#### 2.4.3. Mapping NM Dyshomeostasis

Many iron-containing biological compounds can be studied by the physical phenomenon of nuclear magnetic resonance. Despite the presence of unpaired electrons in NM macromolecules, NM-MRI relies on the paramagnetic nature of NM-iron complexes due to its avid iron binding property, allowing its in vivo visualization due to T1-shortening effects (Figure 2, Panel B) [67]. In the year 2006, the study described an MRI technique suitable for the in vivo imaging of NM-containing structures of the CNS [111]. Here, they used a 3T scanner that revealed high signal intensities in the SNpc and the LC. They demonstrated a marked signal intensity reduction in PwPD compared to HCs [111]. After this seminal study, several research groups developed different NM-MRI sequences to improve spatial resolution and evaluated their respective clinical utility. In this way, NM-MRI has been used as a surrogate marker to assess DA neuron loss, particularly in PD and other neurodegenerative movement disorders [112,113,114]. Table S2 gives an overview of current NM-MRI hardware setups, acquisition parameters, sequences, and study cohorts, including all high-quality studies that evaluated the diagnostic performance of NM-MRI in differentiating PwPD from HCs [115,116,117,118].

#### 2.4.4. NM-Weighted Neuroimaging in PwPD

Systematic reviews and meta-analyses have shown robust reliability for NM-MRI-derived sensitivity and specificity values for differentiating PwPD from HCs [8,119]. The first meta-analysis from 2019 demonstrated a sensitivity of 0.82% (95% CI, 0.74–0.87) and a specificity of 0.82 (95% CI, 0.73–0.89) [119]. In contrast, a second meta-analysis defined their inclusion criteria differently, included newer studies, and substantially increased the number of individuals [8]. Here, the pooled diagnostic performance increased to a significantly higher sensitivity of 89 ± 3% (SD) and a similar specificity of 83 ± 5% (SD). These findings were further supported by more recent studies [115,116,117]. A meta-regression analysis identified the disease duration as a significant covariate, illustrating an overall higher diagnostic accuracy in patients with disease durations ≥5 and even more pronounced ≥10 years [8]. NM-weighted neuroimaging has also been used successfully to compare PwPD, their respective subtypes, and differential diagnoses [120,121,122]. Two studies illustrated the promising application of NM-MRI in differentiating patients with ET and tremor-dominant PwPD [112,123]. However, further research is needed for the reliable differentiation of different forms of parkinsonism among other movement disorders to translate NM-MRI into the diagnostic workup of patients in clinical practice. Based on our literature research, NM-MRI in the study of PwPD will be of outstanding importance. Here, the discrimination of PwPD compared to HCs has been shown repeatedly. MRI scanners from several vendors and different hardware setups (e.g., using different head coils) were used across several studies with similar results, which indicates the high reliability of NM-weighted neuroimaging. However, all studies were performed on high-field (≥3 T) MRI scanners because lower static magnetic field strengths do not achieve sufficient image resolution for subsequent analyses. Interestingly, a recent meta-regression analysis assessed how technical factors influence the diagnostic performance of NM-MRI in PwPD [8]. Technical factors such as a slice thickness ≥2 mm, three-dimensional image acquisitions, (semi-)automated segmentation algorithms, and NM-MRI-derived volumes instead of signal intensities as outcome measures were associated with a markedly improved diagnostic performance [8].

Whether NM-MRI will follow a similar role in the differential diagnosis of other movement disorders will be the subject of future research. This is of particular importance as mitochondrial dysfunction might not play an equally prominent role in other movement disorders. The well-known posteroanterior progression of nigral neurodegeneration can be recapitulated by NM-MRI signal loss, making it a promising tool for the further investigation of prodromal PD stages and the longitudinal evaluation of PwPD. Longitudinal changes of NM-MRI signals in PwPD have demonstrated significant correlations between SNpc signal changes and SNpc volume loss, disease severity, and disease duration [124]. The above-mentioned technical recommendations could further improve diagnostic accuracy (PwPD vs. HCs) by investigating the microstructural properties of the SNpc [8].

#### 2.4.5. Multimodal Iron- and NM-Weighted Neuroimaging and Their Implications for Pathophysiology-Orientated Studies

To date, dopamine transporter imaging (DAT imaging) is considered the gold standard in evaluating SNpc cell loss. Even though DAT imaging can be considered a proxy of DA denervation, it resembles the end route of interwoven pathophysiological processes in vivo. Iron- and NM-weighted neuroimaging can be a suitable technique for differential diagnosis and tracking clinical progression in PwPD. However, it can also be used as a window to gain pathophysiological insights into the disease mechanisms of PD.

DA-, NM-, and iron-related pathways are tightly linked as they are all involved in DA synthesis and degradation [125]. The importance of DAT imaging is undisputed [126]. However, a recent study suggested that the NM-MRI-based segmentation of the SNpc area may yield higher diagnostic accuracies than DAT imaging for the differentiation of PwPD and HCs [127].

One in vivo study highlighted the co-occurrence of DA dysfunction and brain iron deposition, which followed a well-established temporospatial progression pattern. Similar insights have also been derived from the combination of DAT imaging and NM-MRI [125,128,129,130,131,132,133]. A combined NM-MRI and DaTSCAN study demonstrated that the specific binding rate (SBR) of the radiotracer correlated with the SNpc NM-MRI-derived signal loss, suggesting NM-MRI as a DA availability proxy [134]. However, the correlations were not very strong, arguing that there might be technical issues or otherwise unexplained variance between these modalities. In another study, the comparison of the DA transporter density (assessed by the radiotracer ^11^C-PE2I) and NM-MRI-derived measures of nigral depigmentation did not reveal any association [132]. In this sense, more research needs to be done to unravel the relation between NM-MRI and specific radiotracer studies of the DA system. Here, another study demonstrated that SNpc NM-MRI-derived signal loss correlated with the motor symptoms severity (as measured by the MDS-UPDRS-III scale) but not with the striatal SBR in DaTSCAN imaging. Conclusively, the authors suggested that NM-MRI could also be a valuable alternative for clinical monitoring of PwPD patients and may resemble PD-specific pathology to a greater extent [134].

Denervation of terminal DA axons initially occurs in the sensorimotor, then the associative, and finally, the limbic area of the striatum before reaching the SNpc [125,135]. Here, the DA denervation precedes alterations in iron- and NM-deposition. DAT-, iron-weighted-, and NM-weighted neuroimaging showed a high regional overlap [125,135]. The former contradicts the negative results of previous works [128]. However, these results highlight the connection between iron, DA, and NM of the SNpc across the individual disease course. Another multimodal neuroimaging study has also stressed these findings: Here, NM-MRI signal loss correlates with functional surrogate outcomes of the DA system, such as altered DA release in the dorsal striatum or cerebral blood flow in the SNpc [65]. These findings highlight the in vivo link between neuronal activity and SNpc NM [65].

Despite the promise of iron-sensitive imaging in tracking different pathophysiologic pathways, results have to be considered with caution, as iron deposition in areas of neuronal cell loss and astrocytic gliosis could correspond to an epiphenomenon and not necessarily a causative factor, as shown in other diseases such as Huntington’s disease [107]. Two neuroimaging studies on different magnetic field strengths did not correlate between NM-MRI-derived and iron-weighted imaging-derived nigrosome visibility [66,136]. However, whether methodological issues or diverging disease mechanisms between iron deposition and NM loss are responsible for these findings will be the subject of future studies. In summary, NM-MRI can enhance the robustness of other neuroimaging modalities by improving the delineation of the SNpc [137]. These observations finally led to the development of an NM-MRI-derived probabilistic atlas to reduce study disparities in the assessment of SNpc [138].

#### 2.4.6. Mapping Mitochondrial Dysfunction

Several research groups have widely established iron- and NM-weighted neuroimaging in the study of PwPD. In contrast, the study of mitochondrial dysfunction is less well-investigated [7]. This may be because there are no conclusive recommendations on the most suitable method, the availability of necessary hardware, precise image acquisition parameters, or distinct partial aspects of mitochondrial dysfunction worth mapping [7]. In addition, inter-site reliability must be ensured before entering clinical practice, and comparative studies on this topic are currently lacking. The complexity of mitochondrial dysfunction requires concentration on critical end routes, namely bioenergetic disturbances by impairment of OXPHOS and oxidative stress [7]. Different neuroimaging modalities can theoretically map both aspects. The most commonly applied methods are magnetic resonance spectroscopy imaging (MRSI), either ^1^H-MRSI or the heteronuclear counterpart ^31^P-MRSI (Figure 2, Panels C and D). Like MRI, MRSI uses NMR-active nuclei, i.e., nuclei with a non-zero nuclear spin, to generate image contrasts. Following a single acquisition, usually, these biomolecules can be differentiated because they resonate at slightly different frequencies based on their local chemical environments (i.e., the molecular arrangement of NMR-active nuclei and their respective chemical bonds) [139]. MRSI analyzes the chemical composition of tissues in a single voxel or several voxels. Here, the frequency separation of distinct molecules is characterized by their chemical shift (usually displayed on the x-axis normalized to a predefined reference value). Metabolites can be (semi-)quantified by their respective area under the curve, which is proportional to the number of nuclei in that particular chemical environment [139]. Therefore, besides the natural abundance, the T1/T2 relaxation times, and the relative sensitivity of the NMR-active nuclei, the molecular structures play an important role in facilitating detection [139]. Certain metabolites are of particular importance within the scope of mapping mitochondrial dysfunction. ^1^H-MRSI has been used in PwPD for the in vivo measurement of lactate as a reliable surrogate marker of impaired OXPHOS [140]. However, advanced MRSI methods via *spectral editing* also allowed the measurement of glutathione levels, a metabolite necessary for neurons to cope with oxidative stress [141,142]. Interestingly, it has already been demonstrated that this method can be used to map therapeutic interventions [25,142]. ^31^P-MRSI offers the possibility to map high-energy phosphorus-containing metabolites (HEPs), such as *nicotinamide adenine dinucleotide* (NAD), *phosphocreatine* (PCr), and ATP [143]. Recent research in schizophrenia has already illustrated that state-of-the-art imaging facilities can distinguish between NAD+ and NADH, a suitable surrogate marker to assess complex I dysfunction [144]. ATP and PCr form a dynamic equilibrium. As OXPHOS accounts for the majority of generated ATP molecules, these HEPs serve as a surrogate marker for the overall bioenergetic state and specifically for complex V of the ETC. In particular, dynamic assessments of the ATP synthesis via OXPHOS can be achieved by combining ^31^P-MRSI and distinct magnetization transfer imaging protocols [145]. However, the need for high magnetic field strengths (≥3 T), the implementation costs, and the subsequent unavailability of ^31^P-MRSI-required hardware currently hinder the widespread clinical applicability. Concerning the measurement of single ETC complexes, the in vivo-assessment of brain oxygen consumption may provide further insights into complex IV activity [146,147]. Most neuroimaging methods for studying brain oxygen consumption rely on indirect measures that feed into physiological models. In total, three approaches have been proposed for this purpose so far. The first approach is quantitative BOLD imaging, which combines standard fMRI with perfusion imaging to disentangle the mixed BOLD signal. The other methods are *T2 relaxation under-tagging* (TRUST) and *susceptibility-based oximetry* (SBO), which employ different imaging-generation contrast but mainly rely on the same physiological models to estimate in vivo brain oxygen consumption [7]. Similar insights can be derived from ^17^O-MRSI- or PET-based methods (also to investigate complex I activity by ^18^F-BCPP-EF) but require sophisticated hardware setups [148]. The investigation of oxidative stress can be considered mainly preclinical imaging. A promising modality, which does not require the administration of contrast agents, runs on standard-MRI hardware, and offers high spatial resolution, is *QUEnch-assiSTed MRI* (QUEST-MRI) [149]. It indirectly allows one to measure the amount of paramagnetic reactive oxygen species via the treatment-induced change in the in vivo relaxation rate. So far, QUEST-MRI has only been used in rodent models and healthy control subjects [150,151]. Further neuroimaging approaches to map oxidative stress are also feasible with non-MRI-based techniques, such as specific PET radiotracer studies or optical imaging (i.e., via broadband near-infrared spectroscopy, bNIRS) [152]. However, further steps are still needed to enable clinical applicability, including the standardization of methods and further experimental validation. Unfortunately, most of the abovementioned methods have not yet been applied in PwPD, and comparative studies are widely lacking. To our knowledge, only one neuroimaging study has been published investigating the role of subcortical iron deposition and mitochondrial dysfunction [70]. Here, a multiple linear regression model has demonstrated a significant correlation between increased iron deposition in the basal ganglia and the respective subcortical bioenergetic state (i.e., measured by HEPs) [70]. For an extensive review of neuroimaging-based approaches to map OXPHOS and oxidative stress, we kindly refer the reader to our recently published article [7].

## 3. Conclusions

The causality and the temporal dynamics of predominant disease mechanisms in PwPD are widely unclear. For example, it remains elusive whether oxidative stress leads to ETC complex disturbances or vice versa [5]. The same holds true for the involvement of iron or NM within the scope of mitochondrial dysfunction and the individual PD disease course. However, distinct molecular mechanisms’ causal and temporal aspects are crucial for understanding PD pathophysiology and gaining deeper insights for developing targeted DMTs. Multimodal neuroimaging studies on mitochondrial impairment in PwPD are still scarce and lack intra-site reliability. However, iron- and NM-weighted neuroimaging may be particularly suitable for combination with assessments of mitochondrial dysfunction. Specific methodological improvements and technical considerations can be considered a significant prerequisite for designing (adaptive) target engagement clinical trials and may substantially foster the development of individualized therapies in PwPD [153,154]. In contrast, current neuroimaging studies often lack reliable reference values based on extensive cohort studies, and the widespread use of quantitative neuroimaging methods is desirable. In addition, longitudinal studies are needed to identify the temporal dynamics of proposed disease mechanisms. The latter is of outstanding importance to enhancing our current understanding of PD and identifying patients that might benefit the most from pathophysiology-targeted therapies. This idea also extends to the treatment of prodromal study participants, which could further improve the success of neuroprotective treatment strategies.

## Data Availability

Not applicable.

## Supplementary Materials

The following supporting information can be downloaded at: https://www.mdpi.com/article/10.3390/ijms232213678/s1, Table S1: Overview of current iron-weighted neuroimaging studies evaluating the diagnostic performance in differentiating PwPD from HCs. Table S2: Overview of current NM-weighted neuroimaging studies evaluating the diagnostic performance in differentiating PwPD from HCs.

## Abbreviations

^31^P-MRSI: ^31^phosphorus magnetic resonance spectroscopy imaging; arb. units: arbitrary units; aSyn: alpha-Synuclein; ATP: adenosine triphosphate; bNIRS: broadband near-infrared spectroscopy; BOLD: blood-oxygen-level dependent; CI: confidence interval; CNS: central nervous system; DA: dopamine/dopaminergic; DAT: dopamine transporter (imaging); DaTSCAN: dopamine transporter scintigraphy; DMT: disease-modifying treatments; ETC: electron transport chain; FOV: field-of-view; HCs: healthy controls; HEPs: high-energy phosphorus-containing metabolites; iP: inorganic phosphate; LC: locus coeruleus; MDS-UPDRS: Movement Disorders Society Unified Parkinson’s Disease Rating Scale; MPTP: 1-methyl-4-phenyl-1,2,3,6-tetrahydropyridine; MRI: magnetic resonance imaging; MRSI: magnetic resonance spectroscopy imaging; NAD: nicotinamide adenine dinucleotide; NBIA: neurodegeneration with brain iron accumulation; NM: neuromelanin; NMR: nuclear magnetic resonance; OXPHOS: oxidative phosphorylation; PCr: phosphocreatine; PD: Parkinson’s disease; PET: positron emission tomography; PINK1: PTEN-induced kinase 1; ppm: parts-per-million; PwPD: patients with Parkinson’s disease; QSM: quantitative susceptibility mapping; QUEST-MRI: QUEnch-assiSTed MRI; ROS: reactive oxygen species; SBO: susceptibility-based oximetry; SBR: specific binding rate; SD: standard deviation; SN: substantia nigra; SNpc: substantia nigra pars compacta; SWI: susceptibility-weighted imaging; TRUST: T2 relaxation under-tagging.
