# Supplementary material for: Iron- and Neuromelanin-Weighted Neuroimaging to Study Mitochondrial Dysfunction in Patients with Parkinson’s Disease
Source: Int J Mol Sci. 2022 Nov 8;23(22):13678. doi: 10.3390/ijms232213678 (PMC9696602; doi:10.3390/ijms232213678)
Supplement: Supplementary file 1 [file ijms-23-13678-s001.zip › Table_S1.pdf]

Author (year)	Study Type	Study Cohort/ Demographics	Scanner Type	Sequence/ Parameters	Target Region	Outcome Measures	Segmentation Method	Results	Conclusion
R2*/T2*									
Langley et al., 2019 [1]	retro	PwPD: 79<br>HCs: 78	Siemens, Prisma, 3T<br><br>GE Healthcare, Signa HDxT, 3T	Prisma<br>*Sequence:* GRE<br>*Dimension:* n/a<br>*Channels:* 64<br>*TR:* 50 ms<br>*6 TEs:* 4.92 + 4.92 ms*<br>*Flip angle:* n/a<br>*Slices:* n/a<br>*Thickness:* n/a<br>*Voxel size:* n/a<br>*Matrix:* 128 x 128 x 56 mm<br>*FOV:* 220 x 220 mm<br>*Time:* n/a<br><br>Signa:<br>*Sequence:* GRE<br>*Dimension:* 3D<br>*Channels:* 8<br>*TR:* 59.3 ms<br>*16 TEs:* 2.7 + 2.9 ms*<br>*Flip angle:* 12°<br>*Slices:* n/a<br>*Thickness:* 1 mm<br>*Voxel size:* 1.72 mm<br>*Matrix:* 128 x 128 x 56 mm<br>*FOV:* n/a	SN	Comparing iron deposition between PwPD and HC	Manual	Sens: 80%<br>Spec: 71%	Mean R2* in the SNpc defined by neuromelanin-sensitive MRI is significantly increased in PwPD.

				Time: n/a					
Pyatigorskaya et al., 2018 [1]	pro	PwPD: 36<br>HCs: 20	Siemens, Trio, 3T<br><br>Siemens, Magnetom, 7T	Trio, 3T:<br>*Sequence:* GRE<br>*Dimension:* n/a<br>*Channels:* n/a<br>*TR:* 9000 ms<br>*6 TEs*: 24-94 ms*<br>*Flip angle:* 90°<br>*Slices:* n/a<br>*Thickness:* n/a<br>*Voxel size:* 2 mm (isotropic)<br>*Matrix:* n/a<br>*FOV:* n/a<br>*Time*: n/a<br><br>Magnetom, 7T:<br>*Sequence:* T2*WI<br>*Dimension:* 3D<br>*Channels:* 8<br>*TR:* 2180 ms<br>*TE:* 29 ms<br>*Flip angle:* 65°<br>*Slices:* 40<br>*Thickness:* n/a<br>*Voxel size:* 0.5 mm (isotropic)<br>*Matrix:* 512 x 408 mm<br>*FOV*: 192 mm<br>*Time:* n/a	SN	Discriminating PwPD from HCs	Manual	Sens: 82%<br>Spec: 54%	Decrease in SN volume on T2* images.

R2* and QSM									
Li et al., 2019 [1]	retro	PwPD: 28<br>HCs: 28	Siemens, Trio Tim, 3T	*Sequence:* Spoiled multi-echo GRE<br>*Dimension:* 3D<br>*Channels:* 12<br>*TR:* 60 ms<br>*8 TEs:* 6.8 + 6.6 ms*<br>*Flip angle:* 15°<br>*Slices:* 96<br>*Thickness:* 2 mm<br>*Voxel size:* 0.63 mm (isotropic)<br>*Matrix*: n/a<br>*FOV:* 240 x 180 mm<br>*Time:* n/a	SN	Discriminating PwPD from HCs	Manual	AUC: 0.89	First and second order radiomics features were extracted from QSM and R2* maps.<br>First- and second order radiomics features derived from QSM successfully distinguish PwPD from HCs.These features outperformed R2* texture analysis.<br><br>Second-order radiomics texture features were more accurate and sensitive than first order radiomic texture features.
QSM									

Cheng et al., 2019	retro	PwPD: 87<br>HCs: 77	GE Healthcare, Signa HDxT, 3T	*Sequence:* GRE T2*-WI<br>*Dimension:* 3D<br>*Channels:* 8<br>*TR:* 59.3 ms<br>*16 TEs:* 2.7 + 2.9 ms*<br>*Flip angle:* 12°<br>*Slices*: 136<br>*Thickness:* n/a<br>*Voxel size:* 0.86 x 0.86 x 1 mm<br>*Matrix:* 256 x 256 mm<br>*FOV:* 220 x 220 mm<br>*Time:* 10:30 min	SN	Discriminating PwPD from HCs	Manual	Sens: 89%<br>Spec: 87%<br>AUC: 0.96<br>Acc: 0.88	Radiomic features of the nigrosome-1 could be useful in the diagnosis of PwPD and could serve as a surrogate marker for the *swallow tail sign.*
Kim et al., 2018 [1]	retro	ESPwPD: 38<br>HCs: 35	Siemens, Skyra, 3T	*Sequence:* Oblique coronal multiecho data image combination (MEDIC)<br>*Dimension*: 3D<br>*Channel:* 32<br>*TR:* 88 ms<br>*6 TEs:* 11.1 + 11.1 ms*<br>*Flip angle:* 10°<br>*Slices:* 28<br>*Thickness:* 1 mm<br>*Voxel size:* 0.5 x 0.5 x 1 mm	SN	Discriminating PwPD from HCs	Semi-automated	Sens: 100%<br>Spec: 100%	High-spatial-resolution QSM combined with histogram analysis can improve the diagnostic accuracy of ESPwPD.

				Matrix: 384 x 384 mm<br>FOV: 192 x 192<br>Time: 7:30 min					
Ren et al., 2021	retro	PwPD: 95<br>HCs: 95	Philips, Ingenia, 3T	*Sequence:* SWI<br>*Dimension:* n/a<br>*Channel*: n/a<br>*TR:* 20 ms<br>*TE:* 27 ms<br>*Flip angle:* 15°<br>*Slices:* n/a<br>*Thickness:* 2 mm<br>*Voxel size:* n/a<br>*Matrix*: 284 x 230 mm<br>*FOV*: 220 mm<br>*Time:* n/a	SN	Discriminating PwPD from HCs	Manual	AUC: 0.81	Moderate negative correlation of radiomic features with HY stage.
Shahmaei et al., 2019 [1]	retro	PwPD: 30<br>HCs: 15	Siemens, Tim Trio, 3T	*Sequence:* GRE T2*<br>*Dimension:* 3D<br>*Channels:* 32<br>*TR:* 38 ms<br>*6 TEs:* 4-41.8*<br>*Flip angle:* 15°<br>*Slices:* n/a<br>*Thickness:* 1.5 mm<br>*Voxel size:* n/a<br>*Matrix*: 256 x 256 mm<br>*FOV*: 256 x 256<br>*Time:* 9:00 min	SN,<br>RN,<br>Globus pallidus, and Thalamus	Discriminating PwPD from HCs	Manual	<u>SN</u><br>Sens: 100%<br>Spec: 93%<br><br><u>RN</u><br>Sens: 80 %<br>Spec: 100 %<br><br><u>Globus pallidus:</u><br>Sens: 90 %<br>Spec: 86.7 %<br><br><u>Thalamus:</u><br>Sens: 73 %<br>Spec: 66 %	The QSM values had a significant association with disease stage in all investigated nuclei.

Sjostrom et al., 2017 [1]	retro	PwPD: 62<br>PwPSP: 15<br>PwMSA: 11<br>HCs: 14	Siemens, Skyra, 3T	*Sequence:* high-resolution GRE SWI sequences with phase and magnitude data<br>*Dimension:* 3D<br>*Channels:* 20<br>*TR:* 28 ms<br>*TE:* 20 ms<br>*Flip angle*: 15°<br>*Slices:* n/a<br>*Thickness:* n/a<br>*Voxel size:* 0.86 x 0.86 x 1.5 mm<br>*Matrix:* n/a<br>*FOV:* n/a<br>*Time*: n/a	SN and RN	Discriminating PwPD from PwPSP, PwMSA, and HCs	Manual	PwPD vs. HCs (SN):<br>AUC: 0.71	SN susceptibility was increased in PwPD compared to HCs.<br><br>RN susceptibilty may help in the differentiation of parkinsonian disorders.
Takahashi et al., 2018 [1]	pro	PwPD: 39<br>HCs: 25	GE Healthcare, Discovery MR750, 3T	*Sequence:* flow-compensated multiecho spoiled GRE<br>*Dimension:* 3D<br>*Channels:* 32<br>*TR:* 40.4 ms<br>*6 TEs:* 13 + 4.72 ms*<br>*Flip angle:* 10°<br>*Slices:* n/a<br>*Thickness:* 2.4 mm<br>*Voxel size:* n/a	SN	Discriminating PwPD from HCs	Manual	AUC: 0.68	The QSM value was significantly higher in the PwPD compared to HCs.

				Matrix: 384 x 256 mm<br>*FOV:* 220 x 220 mm<br>*Time*: 4:30 min					
Tan et al., 2021	pro	PwPD: 47<br>HCs: 16	Siemens, Skyra, 3T	*Sequence:* GRE T2*-WI<br>*Dimension:* 3D<br>*Channels:* 32<br>*TR:* 53 ms<br>*6 TEs:* 5.3 / 7.5/14.8/20.0/ 26.4 ms*<br>*Flip angle*: n/a<br>*Slices:* n/a<br>*Thickness:* 2 mm<br>*Voxel size:* 0.9 x 0.9 x 2 mm<br>*Matrix:* 256 x 192 mm<br>*FOV:* 230 x 172 mm<br>*Time:* 7:18 min	SN, Caudate, Putamen, and Globus pallidus	Discriminating PwPD from HCs	Manual	AUC: 0.88	Susceptibility value was increased in PwPD in all ROIs except the caudate.<br><br>There was a significant association between the MDS-UPDRS-III score and susceptibility value in the putamen.

Supplementary Table S1. Overview of current iron-weighted neuroimaging studies evaluating the diagnostic performance in differentiating PwPD from HCs. Here, we included only high-quality studies from the last five years (2017 to 2022). [1] included in the systematic review and meta-analysis from Cho et al. (2021). *Number° TE: Number of echoes acquired in a given TR and initial TE +/- Number° (Time echo intervals). 3D: three-dimensional. Acc: accuracy. AUC: area under the curve. ESPwPD: early-stage patients with Parkinson's disease. GRE: gradient-repeat echo. HCs: healthy controls. HY: Hoehn and Yahr. MDS-UPDRS-III: Movement Disorders Society Unified Parkinson's Disease Rating Scale subscore III. MRI: magnetic resonance imaging. n/a: not available. pro: prospective. PwMSA: patients with multiple systems atrophy. PwPD: patients with Parkinson's disease. PwPSP: patients with progressive supranuclear palsy. QSM: quantitative susceptibility mapping. retro: retrospective. RN: red nucleus. ROI: region of interest. Sens.: sensitivity. SN: substantia nigra. SNpc: substantia nigra pars compacta. Spec.: specificity. TE: echo time. TR: repetition time. WI: weighted imaging.
