# Supplementary material for: Iron- and Neuromelanin-Weighted Neuroimaging to Study Mitochondrial Dysfunction in Patients with Parkinson’s Disease
Source: Int J Mol Sci. 2022 Nov 8;23(22):13678. doi: 10.3390/ijms232213678 (PMC9696602; doi:10.3390/ijms232213678)
Supplement: Supplementary file 1 [file ijms-23-13678-s001.zip › Table_S2.pdf]

Author (year)	Study Type	Study Cohort/ Demographics	Scanner Type	Sequence/ Parameters	Target Region	Outcome Measures	Segmentation Method	Results	Conclusion
Fabbri et al., 2017[1]	retro	LSPwPD: 13<br>ESPwPD: 12<br>HCs: 10	Philips, Achieva, 3T	*Sequence:* Modified T1-w fast spin<br>*Dimension:* 2D<br>*Channel:* 12<br>*TR:* 633 ms<br>*TE:* 10 ms<br>*Flip angle:* n/a<br>*Slices:* 20<br>*Thickness:* 2.5 mm<br>*Voxel size:* 0.40 x 0.40 mm<br>*Matrix:* 548 × 474 mm<br>*FOV*: 220 mm<br>*Time:* 8:00 min	SN	Discriminating LSPwPD from ESPwPD	Semi-automated	Sens: 75%<br>Spec: 92%<br>AUC: 0.86	NM Signal decreased in LSPwPD compared to ESPwPD and HCs.<br><br>Weak correlation with HY and MDS-UPDRS-III.
He et al., 2021	pro	ESPwPD: 40<br>HCs: 40	Philips, Ingenia, 3T	*Sequence:* Seven-echo SWI sequence with an Magnetization Transfer pulse<br>*Dimension:* 3D<br>*Channels:* 15<br>*TR:* 62 ms<br>*TE:* 7.5 ms<br>*Flip angle:* 30°<br>*Slices:* 64<br>*Thickness:* 2 mm<br>*Voxel size:* n/a<br>*Matrix:* 384 x 144 mm<br>*FOV:* n/a	SN and parabrachial nuclei	Discriminating ESPwPD from HCs	Semi-automated	NM complex volume:<br>AUC: 0.96<br><br>Total SN volume:<br>AUC: 0.79<br><br>Combination of NM complex volume and SN total volume:<br>AUC: 0.97	NM complex and SN volumes had lower signal intensities; the iron content was higher in the SN for PwPD.<br><br>Subjects with bilateral loss of the nigrosome-1 had the highest iron content.

				Time: 4:47 min					Negative correlation between SN volume and MDS-UPDRS-III.
Le Berre et al., 2019[1]	retro	PwPD: 74<br>HCs: 74	Siemens, Prisma, 3T<br><br>Philips, Achieva, 3T	Siemens:<br>*Sequence:* T1-WI-FSE modified with additional spectral presaturasion IR<br>*Dimension:* 2D<br>*Channels:* 32<br>*TR:* 600 ms<br>*TE:* 12 ms<br>*Flip angle:* n/a<br>*Slices:* 3<br>*Thickness:* n/a<br>*Voxel size:* n/a<br>*Matrix*: 512 x 359 mm<br>*FOV*: 220 x 220 ms<br>*Time*: 7.15 min<br><br>Philips:<br>*Sequence:* T1-WI-FSE modified with additional spectral presaturasion IR<br>*Dimension:* 2D<br>*Channels:* 32<br>*TR:* 688 ms	SN	Discriminating PwPD from HCs	Automated and Manual	Automated segmentation<br>AUC: 0.94<br><br>Manual segentation:<br>AUC: 0.93	Similar AUC following automated and manual segmentation.<br><br>High AUC in differentiating PwPD from HCs.

				TE: 15 ms<br>*Flip angle:* n/a<br>*Slices:* n/a<br>*Thickness:* 3 mm<br>*Voxel size:* 0.43 x 0.43 x 3 mm<br>*Matrix*: n/a<br>*FOV*: n/a<br>*Time:* 7:46 min					
Oshima et al., 2021	pro	PwPD: 25<br>HCs: 49	Siemens, Skyra, 3T	*Sequence:* DANTE T1-Space<br>*Channels:* 32<br>*Dimension:*<br>*TR:* 1000 ms<br>*TE:* 11 ms<br>*Flip angle:* variable<br>*Slices:* 256<br>*Thickness:* n/a<br>*Voxel size:* 0.56 mm (isotropic)<br>*Matrix:* n/a<br>*FOV:* 180x180.<br>*Time:* 5:44 min<br><br>*DANTE pulse*<br>*Flip angle:* 10°<br>148 total pulses<br>*Duration:* 0.08 ms<br>*Spoiler gradient area:* 167.24 ms.	SN	Discriminating PwPD from HCs	Manual	Native space:<br>AUC: 0.83<br><br>MNI space:<br>AUC. 0.72<br><br>Hyperintense areas in native space:<br>AUC: 0.93<br><br>Hyperintense areas in MNI space:<br>AUC 0.92<br><br>Without significant differences between GRE and DANTE.	DANTE T1-SPACE showed significantly higher contrast ratios and larger hyperintense areas than T1-SPACE.<br><br>On DANTE T1-SPACE, HC showed significantly higher contrast ratios and larger hyperintense areas than PwPD.<br><br>No correlation with MDS-UPDRS-III.

Zorzenon et al., 2021	pro	PwPD: 41<br>HCs: 21	Philips, Achieva, 3T	*Sequence*: T1-w TFE<br>*Channels:* 32<br>*Dimension:* 3D<br>*TR:* 7.8 ms<br>*TE:* 3.7 ms<br>*Flip angle:* 8°<br>*Slices:* 320<br>*Thickness:* n/a<br>*Matrix:* 240 x 240<br>*Voxel size:* 1 mm (isotropic)<br>*FOV:* n/a<br>*Time:* 3:08 min.	SN and nigrosome-1	Discriminating PwPD from HCs	Manual	<u>Experts:</u><br>Sens: 90%<br>Spec: 93%<br><br><u>Non-Experts:</u><br>Sens: 92%<br>Spec: 67%	MRI evaluation of SN and nigrosome-1 has high accuracy for diagnosis of PD.<br><br>The multi-echo sequence may facilitate nigrosome-1 evaluation.<br><br>The diagnostic accuracy was higher for experts.
Prasad et al., 2018[1]	retro	PwPD: 16<br>HCs: 15	Philips, Achieva, 3T	*Sequence:* High resolution T1-w anatomical images<br>*Dimension:* 3D<br>*Channels:* n/a<br>*TR:* 26 ms<br>*TE:* 2.2 ms<br>*Flip angle:* 20°<br>*Slices:* 50<br>*Thickness:* 1 mm<br>*Voxel size:* 0.9 x 0.9 x 1 mm<br>*Matrix:* 512 × 512	SN	Discriminating PwPD from HCs	Manual	<u>SNpc central:</u><br>Sens: 88%<br>Spec: 53%<br>AUC: 0.73<br><br>Similar results between central and lateral SNpc.	Lateral SNpc CNR lower than medial SNpc CNR.<br><br>CNRs correlated with disease duration and MDS-UPDRS-III.

				FOV: 180 x 180 x 50 mm<br>*Time:* 4:19 min					
Pyatigorskaya et al., 2018[1]	pro	PwPD: 36<br>HCs: 20	Siemens,<br>Trio,<br>3T	*Sequence:* NM-Sensitive Axial TSE T1-WI<br>*Dimension:* 2D<br>*Channels:* n/a<br>*TR:* 900 ms<br>*TE:* 15 ms<br>*Flip angle:* 180°<br>*Slices:* n/a<br>*Thickness:* n/a<br>*Voxel size:* 0.4 x 0.4 x 3 mm<br>*Matrix*: n/a<br>*FOV:* n/a<br>*Time:* n/a.	SN	Discriminating PwPD from HCs	Manual	<u>NM signal:</u><br>Sens: 88 %<br>Spec: 80%<br>AUC: 0.88<br><br><u>Volume</u><br>Sens: 100%<br>Spec: 72 %<br>AUC: 0.86	Significant decrease in the NM-defined signals and volumes in PwPD.
Schwarz et al., 2017[1]	pro	PwPD: 39<br>HCs: 30	Philips,<br>Achieva,<br>3T<br><br>GE Healthcare, Discovery MR750,<br>3T	<u>Siemens #1:</u><br>*Sequence:* T1-WI-FSE + spectral presaturation IR<br>*Dimension:* 2D<br>*Channels:* 8<br>*TR:* 688 ms<br>*TE:* 9 ms<br>*Flip angle:* n/a<br>*Slices:* 21<br>*Thickness:* 2.5 mm<br>*Voxel Size:* 0.47 x 0.47 mm<br>*Matrix:* n/a<br>*FOV:* n/a<br>*Time:* 12:00 min	SN,<br>LC, and ventral tegmental area	Discriminating PwPD from HCs	Manual	AUC: 0.92<br><br>NM volumen loss of posterior and whole SNpc.	Reduction of normalized NM volume in PwPD was most pronounced in the posterior SNpc, followed by anterior SNpc and LC.<br><br>Correlation with MDS-UPDRS-III.

				Siemens #2:<br>*Sequence:* T1-WI-FSE + Magnetization Transfer<br>*Dimension:* 2D<br>*Channels:* 8<br>*TR:* 904 ms<br>*TE:* 9 ms<br>*Flip angle:* n/a<br>*Slices:* 12<br>*Thickness:* 2.5 mm<br>*Voxel size:* 0.47 x 0.47 mm<br>*Matrix:* n/a<br>*FOV:* n/a<br>*Time:* 12:34 min<br><br>GE Healthcare:<br>*Sequence:* T1-weighted spin-echo sequence with additional "off-resonance" Magnetization Transfer pulse<br>*Dimension:* 2D<br>*Channels:* 32<br>*TR:* 600 ms<br>*TE:* 10 ms<br>*Flip angle:* n/a<br>*Slices:* 12<br>*Thickness:* 2.5 mm					

				Voxel size: 0.38 x 0.38 mm<br>*Matrix:* n/a<br>*FOV:* n/a<br>*Time:* 9:32 min					
Takahashi et al., 2018 [1]	pro	ESPwPD: 39<br>HCs: 25	GE Healthcare, Discovery MR750, 3T	*Sequence:* T1-WI with spectral<br>*Dimension:* 3D<br>*Channels:* 32<br>*TR:* 550 ms<br>*TE:* 10.49 ms<br>*Flip angle:* 180°<br>*Slices:* n/a<br>*Thickness:* 2.4 mm<br>*Voxel size:* n/a<br>*Matrix:* 256 x 256 mm<br>*FOV:* n/a<br>*Time:* 7:56 min	SN	Discriminating ESPwPD from HCs	Automated (using an in-house segmentation method)	<u>Total SNpc area:</u><br>AUC: 0.81<br><br><u>Dorsolateral SNpc area:</u><br>AUC: 0.78	CNRs showed moderate diagnostic performance.
Takahashi et al., 2022	retro	ESPwPD: 32<br>PwRBD: 15<br>HCs: 24	GE Healthcare, Discovery MR750, 3T	*Sequence:* T1-w FSE<br>*Dimension:* n/a<br>*Channels:* 32<br>*TR:* 550 ms<br>*TE:* 10.49 ms<br>*Flip angle:* 180°<br>*Slices:* n/a<br>*Thickness:* 2.4 mm<br>*Voxel Size:* n/a<br>*Matrix:* 256 x 256 mm<br>*FOV:* 180 mm<br>*Time:* n/a	SN	Discriminating ESPwPD from PwRBD and HCs	Manual	SNpc<br>PwPDs vs. HCs:<br>AUC: 0.76	The NM-derived values were significantly lower in PwPD than in HCs.

Taniguchi et al., 2018[2]	pro	PwPD: 24<br>PwPSP: 11<br>HCs: 10	Philips, Achieva, 3T	*Sequence:* Modified T1-w FSE with spectral pressaturation IR (Similar to Schwartz 2017)	SN	Discriminating PwPD from PwPSP and HCs	Manual and automated	PwPD vs. HCs:<br>Sens: 83%<br>Spec: 100%<br>AUC: 0.92.	Correlation between NM and motor symptoms in PwPD.
Zupan et al., 2019[1]	pros	ESPwPD: 20<br>HCs: 12	Philips, Achieva, 3T	*Sequence:* T1-w spectral pre-saturation with inversion recovery (SPIR) sequence<br>*Dimension:* 3D<br>*Channels:* 32<br>*TR:* 25 ms<br>*TE:* 2 ms<br>*Flip angle:* 20°<br>*Slices:* 60<br>*Thickness:* n/a<br>*Voxel size:* 1 mm (isotropic)<br>*Matrix:* n/a<br>*FOV:* 220 x 185 x 60 mm<br>*Time:* 4:33 min	SN	Discriminating ESPwPD from HCs	Manual and semi-automated	Area:<br>Sens: 92 %<br>Spec: 95 %<br><br>Volume:<br>Sens: 92 %<br>Spec: 90 %	Manual and semi-automated segmentation methods of the SN distinguish between PwPD and HCs reliably.<br><br>Analyses demonstrated high Sens and Spec for both methods.

Supplementary Table S2. Overview of current NM-weighted neuroimaging studies evaluating the diagnostic performance in differentiating PwPD from HCs. Here, we included only high-quality studies from the last five years (2017 to 2022). [1] included in the systematic review and meta-analysis from Cho et al. (2021). [2] included in the systematic review and meta-analysis from Wang et al. (2019). 2D: two-dimensional. 3D: three-dimensional. AUC: area under the curve. CNR: contrast-to-noise ratio. ESPwPD: early-stage patients with Parkinson's disease. FSE: fast spin echo. GRE: gradient-repeat echo. HCs: healthy controls. HY: Hoehn and Yahr. IR: inversion recovery. LC: locus coeruleus. LSPwPD: late-stage patients with

Parkinson's disease. MDS-UPDRS-III: Movement Disorders Society Unified Parkinson's Disease Rating Scale subscore III. MRI: magnetic resonance imaging. n/a: not available. NM: neuromelanin. pro: prospective. PwMSA: patients with multiple systems atrophy. PwPD: patients with Parkinson's disease. PwPSP: patients with progressive supranuclear palsy. PwRBD: patients with REM-sleep behavior disorder. QSM: quantitative susceptibility mapping. retro: retrospective. RN: red nucleus. ROI: region of interest. Sens: sensitivity. SN: substantia nigra. SNpc: substantia nigra pars compacta. Spec: specificity. SWI: susceptibility-weighted imaging. TE: echo time. TFE: turbo field echo. TR: repetition time. W: weighted. WI: weighted imaging.
